# Antifungal Activity against *Fusarium oxysporum* of Botanical End-Products: An Integration of Chemical Composition and Antifungal Activity Datasets to Identify Antifungal Bioactives

**DOI:** 10.3390/plants10122563

**Published:** 2021-11-24

**Authors:** Diego Cárdenas-Laverde, Ricardo Barbosa-Cornelio, Ericsson Coy-Barrera

**Affiliations:** Bioorganic Chemistry Laboratory, Facultad de Ciencias Básicas y Aplicadas, Universidad Militar Nueva Granada, Cajicá 250247, Colombia; u0500822@unimilitar.edu.co (D.C.-L.); rabarbosac@gmail.com (R.B.-C.)

**Keywords:** *Fusarium oxysporum*, botanical extracts, mycelial inhibition, conidial susceptibility

## Abstract

Plants produce various compounds as defensive barriers to naturally control fungal diseases. Among them, vascular wilt caused by *Fusarium oxysporum* is one of the most destructive diseases in crops, causing relevant economic losses. The application of synthetic fungicides is the most used management for this disease. However, this kind of method also involves adverse environmental impacts. Therefore, alternative methods are continuously being developed as a strategy to be involved in integrated pest management programs. Thus, as part of our research on antifungals of plant origin, a group of botanical extracts was assessed for the respective inhibitory effect on mycelium and conidia of *F. oxysporum*. Mycelial growth inhibition was measured in 12-well plates containing amended semi-solid medium, whereas conidial susceptibility was determined through microdilution. The identification of the bioactive compounds among test extracts was performed using an indirect approach, consisting of the integration of chemical composition and antifungal activity datasets through single-*Y* orthogonal partial least squares (OPLS) regression. Results showed that *Piper aduncum* extract was the most potent mycelial growth inhibitor whereas *P. elongatum* exhibited the best effect on conidia susceptibility. The active compounds identified through statistical integration and subsequent isolation were piperaduncin C, asebogenin and (−)-methyllinderatin. These findings indicated that the integrative, indirect approach is useful for the identification of bioactive metabolites from botanical extracts to be further used as biological protective agents against this phytopathogen.

## 1. Introduction

Plants are well-known factories and huge reservoirs of naturally-occurring compounds, many of them used by plants in ecological relationships to be able to relate and subsist [1]. These compounds, usually comprising specialized metabolites (formerly called secondary), are characterized by being biologically active and employed in defensive roles, among others, to maintain a natural balance with the environment, pathogens and predators [2]. Indeed, some of these metabolites are defensive barriers against microbial diseases, especially those caused by soil-borne fungal pathogens [3]. Among them, different Ascomycota fungi, particularly *Fusarium* species, are phytopathogens that affect several economically-important crops. In this group, *Fusarium oxysporum* is one of the most widespread pathogens globally, comprising more than 120 reported *forma specialis* [4]. This fungal phytopathogen can cause many losses through the vascular disease so-called ‘Fusarium Wilt’. Consequently, agronomic practices have been developed for controlling this disease, such as biological control, cultural practices and chemical control. The most common agricultural practice is oriented to chemical control by using synthetic fungicides since such a strategy is versatile and easily adopted [5]. Currently, the most used compounds to control the presence of this phytopathogen are mainly nitrogen-containing heterocyclic compounds, such as azoles, benzimidazoles, dithiocarbamates and dicarboximides [6]. However, this practice leads to environmental damage due to their toxicity [7] but promotes pathogen resistance. In this regard, the acquired resistance by phytopathogens to the available synthetic products makes their control increasingly more difficult [8]. This fact promotes abuse of the application of these fungicides and, consequently, generates their gradual loss of efficacy, causing the accumulation of such substances and altering the dynamics of the populations of soil-living microorganisms [9]. In addition, consumption of fruits and vegetables, previously treated with low or high doses of synthetic fungicides is also a cause of toxicity that impacts human health [10].

Several studies seek eco-friendly strategies to avoid perturbations of environmental dynamics, as well as the health of humans and animals. Alternative methods for the control of diseases have been studied with emphasis on compounds derived from plant sources, such as essential oils and botanical extracts, which can usually have higher safety for consumers and the environment or they can be efficiently incorporated as a part and/or phase of integrated pest management programs [11,12,13]. These plant end-products contain different types of metabolite classes, such as terpenoids (e.g., mono, di, triterpenoids and steroids), alkaloids, and phenolics (e.g., flavonoids, tannins, proanthocyanidins, and other phenolics-like compounds) [14]; however, they are selective and/or have specific roles in different ecological and endogenously biological events, so the exploration of such chemodiversity to look for antifungals from a particular plant source to control the fungal pressure on another plant is continually required through screening procedures [15,16]. Despite the activity exhibited by a botanical end-product, the active principles responsible for antifungal activity are not easily documented or recorded. Nevertheless, it is very important to identify those metabolites responsible for a particular effect in order to link the biological activity to a specific compound, even if the botanical extract would be employed. In this sense, there are several methods to establish their activity, either directly or indirectly. Within direct methods, bio-guided fractionation searches for the activity of depurated fractions from a parent extract to get the final identification of the bioactive components. However, this procedure is time-consuming and expensive [17]. On the other hand, indirect methods might overcome such disadvantages. An emergent indirect method to detect bioactives is based on the statistically-mediated integration of the chemical composition to be related to its biological activity [18,19].

In the present study, as part of our research on antifungals of plant origin, the inhibitory capacity on *F. oxysporum* growth of forty-four botanical end-products from an in-house plant collection was evaluated through mycelial growth on solid medium and conidial susceptibility in liquid medium. From the integration of these chemical and biological datasets, the bioactive constituents against *F. oxysporum* were then detected and identified.

## 2. Results and Discussion

### 2.1. Antifungal Activity of Test Botanical Extracts

Results on *F. oxysporum* mycelial growth inhibition (MGI) percentage at 0.2% (*w*/*v*) of all test extracts (*n* = 44) is presented in Table 1. The MGI covered was ranging from 12% to 95%, involving an average of 48.8% and a median of 54.08% (relative frequency of 0.38). Extracts showing the highest antifungal activity were found to be those derived from *Piper* plants, such as four extracts from *Piper aduncum* and *P. elongatum* (MGI > 76%), which were significantly different from the rest of the test extracts (*p* < 0.05). This result agrees with previous studies since various *Piper* plants are widely studied for their antifungal properties [20], even against *F. oxysporum,* such as *P. hispidum* (essential oil) [21] and *P. nigrum* (essential oil and extracts) [22]. Several specialized metabolites, such as lignans, neolignans, kavapyrones, flavones, flavanones and phenylpropanoids are closely related to *Piper* plants [23]. These compounds are considered to be responsible for various activities, such as antibacterial, antifungal, anti-inflammatory, antiplatelet, insecticide, antioxidant, cytotoxic, antiplasmodic, and antifungal activity against phytopathogens, such as *F. oxysporum* [22,24]. Indeed, some chemical differences depending on the plant part, phenology, environment, and genetic factors have been revealed for some *Piper* plants, influencing the biological activity of a particular plant source [20] and, consequently, expanding the scope of *Piper*-based products as a source of antifungals. 

The most active and significantly different extract was *P. aduncum* (MGI = 95.3%). This plant has exhibited various activities, such as cytotoxic, antimicrobial [25], antifungal [26] and insect repellency [27]. In previous studies conducted on *Fusarium* species, the essential oil from *P. aduncum* showed important fungistatic action on *Fusarium redolens* and *Fusarium solani* [28]. In addition, *P. aduncum* showed activity against other species, such as *Alternaria brassicicoli*, *A. chrysanthemi*, *Aspergillus flavus*, *A. fumigatus*, *Candida albicans*, *Cladosporium herbarum*, *C. sphaerospermum* and *C. cladosporioides* [26,29].

Additionally, among test extracts, the MGI of other botanical end-products from Fabaceae, Lauraceae, Brassicaceae, Bignoniaceae and Burseraceae plants (involving nine extracts) also exhibited relevant inhibitory capacity (MGI > 55%), highlighting *Anadenanthera* *peregrina* and *Nectandra longifolia* leaves-derived extracts by their effect on *F. oxysporum* growth (MGI > 68%). Contrarily, extracts obtained from *Cedrela odorata*, *Senna viarum*, *Tithonia diversifolia*, and *Virola elongata* exhibited low inhibitory activity against *F. oxysporum* (MGI < 30%).

**Figure 1:**
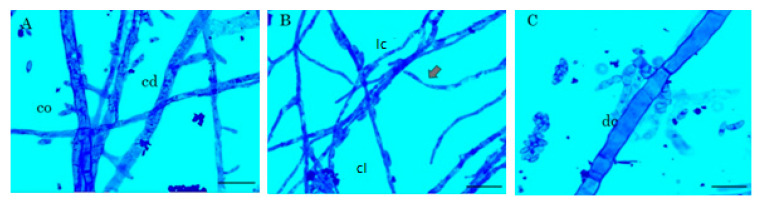
Microscopic visualization of *F. oxysporum* mycelium stained with lactophenol blue. (**A**) Normal growth of *F. oxysporum* mycelium (Control). 2000×. bar 25 μm. (**B**) Treatment with *P. aduncum* leaves-derived extract at 0.2% (*w*/*v*). 2000×. 25 μm. (**C**) Treatment with Dithane at 0.2% (*w*/*v*). 2000×. bar 25 μm. Features: (co) conidia, (cd) cytoplasm densification (lc) loss of cytoplasm, (cl) cross-linking of hyphae, (arrow) reduction and lysis, (dc) disintegration of cytoplasm.

The microscopic effect of the most active extract (i.e., *P. aduncum* leaves, *Pa*L) on *F. oxysporum* mycelium was also recorded. In the control treatment, normal mycelial growth and shape was observed, including a normal spore production (Figure 1A). In contrast, dithane caused visible damage in fungal structures (Figure 1C), particularly related to hyphal damage and cytoplasm disintegration [30]. However, dithane also stimulated the overproduction of viable spores as a response to the fungicide in comparison to the control [31]. This outcome coincided with previous reports since the active principle mancozeb has been reported as a sporulation promoter on *F. oxysporum* [32]. Thus, despite the important inhibitory activity on mycelial growth, the sporulation stimulation by dithane could be considered an adverse effect on controlling the pathogen proliferation at the field level. 

In the case of *P. aduncum*, the effect was more evident even in comparison to the other test extracts and positive control, since several damages on fungal structures were observed. *Pa*L-derived extracts caused loss of cytoplasm, cell reduction and lysis (Figure 1B), which suggested that the chemical components of this extract would target cell wall or cell membrane. Other effects were also detected, such as agglomeration, hyphal cross-linking, and considerable increase of vacuoles [33] and, finally, the spore production was reduced and the effect was found to be fungicide. These effects can be rationalized due to this active extract could contain lignans and flavonoids, common phytoconstituents reported for *P. aduncum* [34]. Such phenolic compounds can disrupt the cell wall of pathogenic fungi, promoting its osmotic lysis [35,36], and other subsequent fungal malfunctions and anomalies, as reported previously for phenolic-rich extracts and individual phenols against phytopathogenic fungi [37,38]. 

On the other hand, the conidial susceptibility on non-germinated *F. oxysporum* conidia suspensions for the entire set of extracts at 0.1% (*w*/*v*) was also evaluated. Since the assessment of conidial susceptibility through microdilution implies an indirect extent of conidial germination inhibition (CGI) of molds [39], it represented another fungal structure to observe the effect of test extracts. However, most extracts (86.4%, *n* = 38) exhibited no CGI at the test dose and they were, therefore, discarded. The remaining six extracts exhibited inhibitory activity, whose results are presented in Table 2, ranging from 15 to 84% GI. The most active extracts were *P. elongatum* leaves (*Pe*L) (CGI = 83.5%) and *T. diversifolia* stems (*Td*S) (CGI = 74%), being significantly different (*p* < 0.05) to the other test extracts. Interestingly, the extracts with high inhibitory capacity on mycelial growth (i.e., *Pa*L and *Pe*S) exhibited low activity on conidial germination (CGI < 25). *Td*F-derived extract exhibited low activity on both mycelial and conidial susceptibility assays (<20%). 

The outcome from both methods (i.e., mycelial growth inhibition and conidial susceptibility) cannot be compared, due to two factors, i.e., the characteristics (e.g., cell wall composition or shape) of each fungal form [40] and exposition manner of extracts in each assay (i.e., submerged cultures vs. agar surface) [41], promoting considerable differences between them. However, *F. oxysporum* mycelium appeared to be more susceptible to most extracts under the conditions of the in-vitro test on amended solid medium, although only six extracts exhibited an in-vitro effect on conidia susceptibility under the submerged culture characteristics, possibly by solubility issues and/or selectivity to this fungal form. In this sense, the activity evaluation on these two fungal forms is very important to be evaluated as a combined/complementary information, considering the corrective aim of mycelial growth inhibition and the preventive scope of conidial germination [42]. In addition, owing to the homogeneity and quality of inoculum the major challenge of the microdilution assay for filamentous fungi, microscopic scrutiny of conidial/spore germination is recommended to verify the information provided by the conidial susceptibility indirectly measured by optical dispersion [41]. Accordingly, all six extracts effectively inhibited the conidial germination at different levels after microscopic inspection of treated liquid cultures in comparison to the control. 

### 2.2. Integration of Chemical and Activity Datasets: Indirect Recognition of Bioactives

Chemical composition (based on chromatographic profiles) and antifungal activity (based on MGI) datasets were integrated through supervised statistics to indirectly recognize bioactives within test botanical extracts. They exhibited different profiles as expected, involving 125 differentiable signals. Some extracts shared particular signals having different intensities, but other signals were exclusive for a particular extract. The chromatographic profiles were decomposed into absorbance at 270 nm as a function of time (each 0.64 s) and a data matrix was then built (5064 × 44). The resulting data matrix was employed for the subsequent procedure. Thus, a supervised statistical analysis, by means of single-*Y* orthogonal partial-least squares (OPLS) regression (using MGI as continuous Y-variable), was used to integrate the chemical composition and biological activity of test plant extracts. The resulting statistical model (consisted of one predictive and one orthogonal component) could separate extracts, revealing an adequate explained variance (R^2^_X_ = 0.78, R^2^_Y_ = 0.82) and predictability (Q^2^_Y_ = 0.46).

**Figure 2:**
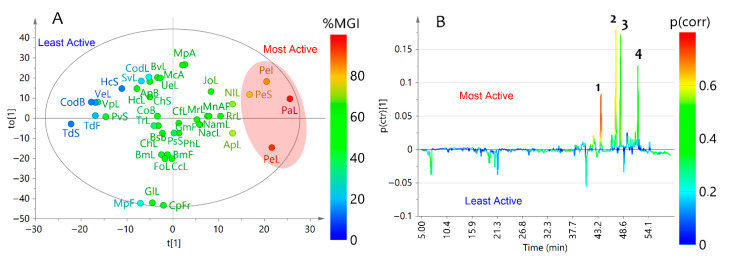
Single-Y orthogonal partial least squares (OPLS) as the integration tool for chromatographic data and mycelial inhibition of *F. oxysporum* (as continuous Y-variable) datasets. (**A**) Scores plot. (**B**) *S*-line plot. Extracts codification is related to the information presented in Table 1.

The resulting scores plot (Figure 2A) showed the discrimination depending on the chemical composition but also according to the MGI data as a continuous *Y*-variable, comprising the first predictive component, t(1), versus the first orthogonal component, to(1), that explain the variations according to the MGI activity (37.2%) and chemical composition (40.8%), respectively. Such Single-*Y*-depending discrimination is intuitively visualized as a heat map representation (0–100% scaling) between high (red) and low (blue) MGI values. This two-factor discrimination was very important for our purpose since the scores plot revealed those extracts discriminated by chemical composition, but the chemical composition related to the MGI activity as a statistically important trend. Once the pattern recognition between datasets was found, the loadings were investigated according to their discriminating influence, using an *S*-line transformation. This *S*-line plot is a tailor-made visualization and interpretation tool for point-to-point decomposed 2D data (e.g., chromatographic profiles under ultraviolet-visible (UV-Vis) detection, infrared (IR) spectra or nuclear magnetic resonance (NMR) spectra) [43,44]. It is very useful to show the covariance loading (*p*(ctr)) along the chromatographic signals (i.e., variables expressed as time values) and colored according to the absolute value of the correlation loading, *p*(corr) [45], providing a linear indication of the correlation intensity. Hence, the single-*Y* OPLS-derived *S*-line plot visualized differences between least active (negative) and most active (positive) extracts (Figure 2B). Consequently, three metabolites (**1**–**3**) were found to influence (*p*(corr) > 0.5) the OPLS-based discrimination between most and least active extracts and, consequently, they were identified as the significant metabolites to the observed MGI. Compound **1** was the most significant metabolite (*p*(corr) = 0.75), and occurred in *P. aduncum* and *P. elongatum* extracts, whereas compound **4** exhibited good reliability but poor correlation loading (*p*(corr) = 0.34). Therefore, compound **4** was not considered a good candidate in the pattern recognition of the inhibitory activity on mycelial growth.

**Figure 3:**
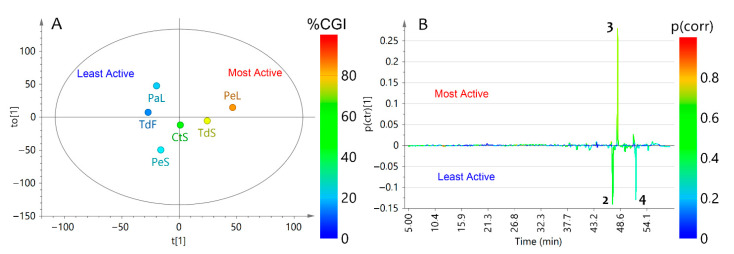
Single-Y orthogonal partial least squares (OPLS) as the integration tool for chromatographic data and conidial susceptibility of *F. oxysporum* (as continuous Y-variable) datasets. (**A**) Scores plot. (**B**) *S*-line plot. Sample codification is related to the information presented in Table 1.

A similar procedure was employed to the SGI results. The corresponding single-Y OPLS model also separated extracts with suitably explained variance (R^2^_X_ = 0.76, R^2^_Y_ = 0.93) and predictability (Q^2^_Y_ = 0.51) and led to the plots presented in Figure 3. Similarly, integration of conidial susceptibility and chemical datasets resulted in an adequate relationship along the t(1) (39.5%) versus to(1) (36.5%). Additionally, the respective *S*-line plot revealed that only compound **3** showed the best correlation (*p*(corr) = 0.70), being the most influencing variable for this SGI assay, whereas compounds **2** and **4** were more related to the least active extracts.

Our findings indicate that a correlation between plant metabolite profiles and antifungal activity datasets can be effectively done through single-*Y* OPLS to recognize patterns under the decomposition of the spectral data into uncorrelated latent variables. OPLS (and even PLS) differs from principal component analysis (PCA) in the maximization of the covariance of independent variables (i.e., spectral data from IR, UV, or MS) as a function of a dependent variable (i.e., biological activity), either continuous or categorical [46]. Consequently, there is an increasing number of studies focused on the application of untargeted metabolomics to integrate the chemical composition and the activity of natural product mixtures [19,47]. Metabolomics-based approaches are actually employed to profile multiple mixture components simultaneously, typically through the application of chromatographic and/or spectroscopical analyses [46]. The use of metabolomics as the source of independent variables to be integrated with a dependent variable is the major advantage of this approach [19]. Indeed, it can also recognize unstable compounds that would be lost upon purification, which is the main challenge during fractionation procedures [48,49]. However, an innate restraint of chemical and biological dataset association is the recognition of false positives, since this integration does not have causal but correlative essence [50]. Therefore, statistically-identified bioactives are required to be fractionated or isolated to validate the correlative recognition [51]. In our case, the process of isolating the compounds with the highest statistical influence on the discrimination of the most active and least active extracts was undertaken in order to validate the correlative finding, since compounds **1–3** represented the best correlation values (*p*(corr) > 0.6).

### 2.3. Isolation and Identification of Antifungal Metabolites

**Figure 4:**
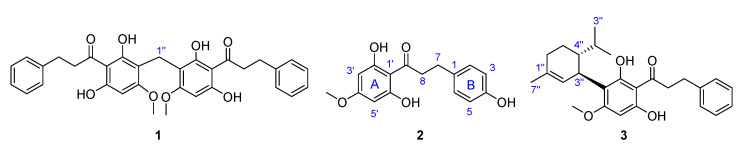
Structures of isolated compounds after OPLS-based recognition.

The OPLS-based integration recognized compounds **1**–**3** as the most influencing metabolites in the antifungal activity from both assays (i.e., mycelial growth inhibition and conidial susceptibility of *F. oxysporum*). To validate this indirect recognition of potential bioactives, these compounds were purified by semipreparative HPLC separation from *Pa*L and *Pe*L-derived extracts and structurally elucidated by nuclear magnetic resonance (NMR). This analysis supported the unequivocal identification of these compounds (i.e., **1**–**3**) as known metabolites since their ^13^C NMR profiles were identical to those reported for piperaduncin C (**1**) [52], asebogenin (**2**) [53] and methyllinderatin (**3**) [54], respectively. In the case of **3**, its optical rotation showed the same sign (i.e., levorotatory) as that reported previously for (−)-methyllinderatin, isolated from leaves of *P. aduncum* [55]. Hence, the structures of these isolated, identified compounds are presented in Figure 4.

The IC_50_ values for the three purified compounds are presented in Table 3. Piperaduncin C (**1**) and asebogenin (**2**) exhibited the most potent antifungal effect through mycelial growth inhibition (IC_50_ = 38.2 and 25.6 μM, respectively), while (−)-methyllinderatin (**3**) showed the lowest activity (IC_50_ = 689.2 μM). In contrast, **3** exhibited the best activity in conidial susceptibility (IC_50_ = 22.3 μM), while **1** and **2** were inactive (>1000).

The three isolated compounds **1**–**3** shared the dihydrochalcone moiety but involved structural differences related to the hydroxyl substituent at ring B (in **2**), and the dimeric and adduct forms, having the particular structural variation on ring A (in **1** and **3**, respectively) (Figure 4). Piperaduncin C (**1**) is a dimeric dihydrochalcone metabolite which exhibited moderate antibacterial activity against *Bacillus subtilis* and *Micrococcus luteus* [52], whereas asebogenin (**2**) (4′-methoxy-2′,4,6′trihydroxydihydrochalcone) exhibited higher activity against these bacteria [52] but also against *Staphylococcus aureus* and methicillin resistant *S. aureus* [56], and antifungal activity against *Candida albicans*, *C. krusei*, *C. parapsilosis* and *Cryptococcus neoformans* (MIC < 15.6 µg/mL) [57]. Finally, (−)-methyllinderatin (**3**) is an interesting monoterpene-dihydrochalcone adduct and exhibited antibacterial activity against *M. luteus* [55]. However, the antifungal activity against *Fusarium* species of these compounds **1**–**3** has not been reported yet, so they are evaluated for the first time in the present study against mycelial growth and conidial susceptibility of *F. oxysporum*. The discovery of these compounds as antifungals was made possible by statistical integration of HPLC-based chemical composition and antifungal activity datasets, confirming that this approach is a valuable tool for identifying antifungals against *F. oxysporum* from a reasonable number of botanical end-products.

## 3. Materials and Methods

### 3.1. Preparation of Botanical Extracts

From an *in-house* collection of native and exotic plants sampled in different places of Colombia (*n* = 34), forty-four botanical extracts were prepared. Plants were collected in Meta, Casanare, Cundinamarca and Guaviare departments (coordinates 3.543, −73.669; 5.190, −72.547; 4.931, −74.008; 2.332, −72.616, respectively) between 2014 and 2017. Voucher specimens are kept at Colombian National Herbarium. The selected healthy plant materials (without visible damage) were separately air-dried and extracted using 96% ethanol at constant shaking speed (120 rpm) using a Heidolph Rotamax 120 platform orbital shaker (Heidolph Instruments GmbH & Co.KG, Schwabach, Germany). The extraction lasted one week with daily removal of the extract-containing solvent and replaced by fresh 96% ethanol. These mixtures were separated by filtration and the resulting solution was concentrated by distillation under reduced pressure at 40 °C using an IKA RV 10 Control rotary evaporator (IKA^®^ RV 10, IKA^®^ Werke GmbH & Co. KG, Staufen, Germany) to afford the raw extracts. Resulting raw extracts per plant from each daily extraction were collected, dried and subsequently stored at −20 °C until use.

### 3.2. In-Vitro Assays against F. oxysporum

*F. oxysporum* LQB-03, a virulent isolate obtained from wilting *Physalis peruviana* plants [58] was used as the test phytopathogen. This isolate has been preserved on Whatman paper # 1 at −20 °C, and further reactivated in potato dextrose agar (PDA) to be used in the antifungal assays. The in-vitro antifungal activity against *F. oxysporum* was evaluated through the inhibition of mycelial growth (12-well plate amended semi-solid medium assay) and conidial susceptibility (96-well plate microdilution assay). Both assays were performed using the respective monosporic culture of the test fungal isolate, developed in PDA (potato-dextrose-agar) medium for 8 days.

#### 3.2.1. Mycelial Growth Inhibition Assay

A micro-scale amended medium protocol was performed following the previously reported procedure [59]. Briefly, 12-well plates (autoclavable glass) and semi-solid medium (1.2 g of PDB (potato-dextrose broth) and 0.5 g of Agar per 100 mL of distilled water) were used. The final volume (for treatments and controls) was 200 μL per well (1-cm diameter). The test concentration (i.e., 0.2% *w*/*v*) was prepared by supplementing fresh semisolid-medium with the required amount of each test dry, raw extract (*n* = 44) (i.e., the residue after extraction without solvent). A stock dispersion was reached by a direct mixture between the semi-solid medium (5 mL) and the required extract (10 mg). This mixture was vigorously stirred until achieving a homogenous dispersion before solidifying. Tween-20 (5%) was used to assist the dispersion of the extracts. The resulting homogeneously amended medium was added into three wells of the 12-well plate. Subsequently, a 1-mm agar-mycelial plug from 5-days actively growing cultures of *F. oxysporum* was inoculated onto the center of each well. Each 12-well plate was placed into a 17-mm Petri dish, under the appropriate conditions of humidity and sterility, and sealed with plastic films. Each trial comprised a randomized design with three replicates for each extract compared to an absolute control (untreated semi-solid medium). Sportak (prochloraz) and Dithane (mancozeb) were employed as positive controls using the same concentration (i.e., 0.2% *w*/*v*). After inoculation and sealing, this assembly was incubated at 25 °C. The assay was concluded once the colony on the negative control covered the whole well (after ca. 48 h). Mean colony area (mm^2^) was, therefore, measured for treated and untreated wells, under processing in the software Image J^®^ using the respective photographic records. The percent mycelial growth inhibition (MGI) was then calculated for each replicate. This calculation was made employing this equation: % MGI = [(absolute control area − treatment area)/absolute control area)] × 100. Finally, a portion of the mycelium after being treated with botanical extracts was removed and stained with Lactophenol Blue. The stained fungal structures were visualized microscopically at 2000× using the Motic BA210 microscope (Motic, Carlsbad, CA, USA).

#### 3.2.2. Conidial Susceptibility Assay

A microdilution protocol was implemented following the previously reported procedure [60]. The assays were carried out in 96-well plates by homogeneously mixing the test extracts in PDB liquid medium and DMSO (>0.2%) at 0.1% (*w*/*v*) as the final dose. Four replicates were adopted for each test extract. To prepare the non-germinated conidial suspensions, 1 mL of sterile distilled water (SDW) was added to a 9-mm Petri dish containing a 5-days actively growing culture of *F. oxysporum*. After that, an aliquot (100 µL) was placed onto the Neubauer chamber to perform the conidia counting. The final inoculum (1 × 10^6^ conidia/mL) was adjusted using the appropriate amount of SDW. The experiment comprised the following mixtures: (1) Plant extract + conidia + PDB medium (treatment); (2) conidia + PDB medium (absolute control); (3) plant extract + SDW (blank 1); (4) PDB medium + SDW (blank 2). Treatment (180 µL) and previously prepared non-germinated conidia dispersion (20 µL) were combined to start the trial. SDW (20 µL) was added instead conidia dispersion for blanks 1 and 2. Prochloraz^TM^ (iprodione) and Dithane FMB^TM^ (mancozeb) were used as positive controls. The resulting optical dispersion (OD) values were taken at 0 and 8 h, using an Elisa EZ Read 2000 analyzer, recording at 600 and 680 nm. Due to the assessment of conidial susceptibility through 8-h microdilution from non-germinated conidial suspension assuming an indirect extent of conidial germination inhibition (CGI) of filamentous fungi [39], we opted to employ this indirect measurement. The calculation of CGI was carried out employing this equation: % CGI = [(OD_cor_ absolute control − OD_cor_ treatment)/OD_cor_ absolute control)] × 100, where OD_cor_ means OD values corrected with blanks 1 and 2. Finally, a portion of the conidia submerged cultures after treated with botanical extracts was removed and stained with Lactophenol Blue. The stained conidial structures were visualized microscopically at 2000× using the Motic BA210 microscope (Motic, Carlsbad, CA, USA) to verify the conidial germination and absence of hyphal structures.

### 3.3. High-Performance Liquid Chromatography Coupled to Diode Array Detector

Chromatographic profiles of test extracts were recorded on a Shimadzu Prominence (Shimadzu Corporation, Kyoto, Japan) equipped with two binary pumps, autoinjector, and a photodiode array detector. Each botanical extract was then dissolved in absolute ethanol (5 mg/mL) and injected (20 µL) into the HPLC system. The separation system consisted of a Synergi C18 column (Phenomenex, Torrance, CA, USA) (4.6 mm × 150 mm, 4 μm), and a combination of solvent A (1% formic acid in acetonitrile (ACN)) and solvent B (1% formic acid in Mili-Q H_2_O). A gradient elution method was used as follows: 0–2 min 0% B, 2–25 min 0% to 50% B, 25–35 min 50% B, 35–50 min 50% to 100% B, 50–55 min 100%, and 55–50 min 100% to 0% B, at 0.7 mL/min. The monitoring wavelength was 270 nm.

### 3.4. Statistical Analysis

A Shapiro–Wilk test was performed to determine the data normality of the inhibition percentage data. Once normality was verified, ANOVA tests were conducted on all samples (type I error, significance value *p* < 0.05) followed by post-hoc Tukey tests to determine the significant differences between data. These statistical tests were performed software R version 3.4.1 (R Foundation, Vienna, Austria).

On the other hand, chromatographic profiles of the test extracts were exported to an ASCII 2D format and a matrix containing point-to-point HPLC data per extract sample was built (5428 × 44). This dataset was aligned in the Matlab^®^ software (Vr2013a) (The Mathworks Inc., Natick, MA, USA). The aligned data were also normalized and autoscaled. These pre-treated chemical data (i.e., chromatographic profiles) were combined with the respective antifungal data (i.e., %MGC or %CGI) to assemble the integrated dataset. The resulting matrix was then imported into the SIMCA software (v 14.0) (Umetrics, Umeå, Sweden) to build the respective models by single-*Y* orthogonal partial least squares (OPLS). The obtained results were visualized by means of the scores and *S*-line plots.

### 3.5. Purification and Identification of Most-Active Compounds by Semipreparative-Scale HPLC

Portions of the *Pa*L and *Pe*L extracts (600 mg) were pre-treated with SPE Strata^®^ C18-U cartridges (55 µm, 70 Å, 500 mg, 6 mL) (Phenomenex, Torrance, CA, USA). The most active microfractions were then collected using an UFLC Prominence system (Shimadzu, Columbia, MD, USA), operated in a semipreparative mode, consisting of a pump (LC-20AD), a column oven (CTO-20AC), a UV/Vis detector (SPD-20AV), an autosampler (SIL-10AP), a fraction collector (FRC-10A) and equipped with a reversed-phase Phenomenex Luna C_18_ column (250 × 10 mm, 5 μm) (Phenomenex, Torrance, CA, USA) at 20 °C. Ten consecutive injections of SPE-depurated extract (500 μL per injection, 80 mg/mL in MeOH) were separated at a flow rate of 3 mL/min using solvents A (1% formic acid in ACN) and B (1% formic acid in H_2_O) using an isocratic elution method. Targeted peaks, according to the OPLS-based recognition, were collected at retention time ranges 43.7–44.2 min (1.8 mg), 46.9–47.5 min (2.5 mg), and 47.9–48.4 min (2.1 mg), to afford pure compounds **1**–**3**, respectively. Structures of isolated compounds were elucidated by ^1^H and ^13^C NMR, through the attached proton test (APT), on an Agilent DD2 600 MHz spectrometer (Bruker, Billerica, MA, USA) using CDCl_3_ as solvent. All shifts are given in δ (ppm) using tetramethylsilane (TMS) as internal standard. Coupling constants (J) in Hz. APT ^13^C NMR data of isolated compounds were identical to that reported for piperaduncin C (**1**) [52], asebogenin (**2**) [53] and (−)-methyllinderatin (**3**) [54,55].

Piperaduncin C (**1**). Amorphous yellowish solid. ^1^H NMR (CDCl_3_, 600 MHz) δ_H_ 10.55 (2H, s, OH-2′), 7.30 (2H, d, *J* = 7.3 Hz, H-4), 7.10–7.18 (8H, m, H-2, H-3, H-5, H-6), 6.15 (2H, s, H-3′), 3.98 (6H, s, CH_3_O-4′), 3.76 (2H, s, H-1″), 3.46 (4H, t, *J* = 7.7 Hz, H-7), 3.06 (4H, t, *J* = 7.7 Hz, H-8). APT ^13^C NMR (CDCl_3_, 150 MHz) δ_C_ 205.7 (C-9 × 2), 165.2 (C-2′ × 2), 161.3 (C-4′ × 2), 158.8 (C6′ × 2), 141.9 (C-1 × 2), 128.6 (C-2, C-6 × 2), 128.3 (C-3, C-5 × 2), 126.0 (C-4 × 2), 106.2 (C-3′ × 2), 104.5 (C-1′ × 2), 92.4 (C-5′ × 2), 56.5 (CH_3_O-4′ × 2), 45.8 (C-7 × 2), 30.4 (C-8 × 2), 15.7 (C-1″ × 1). HRESIMS [M + H]^+^ *m*/*z* 557.2166 (calcd. for C_33_H_33_O_8_, 557.2175).

Asebogenin (**2**). White amorphous solid. ^1^H NMR (CDCl_3_, 600 MHz) δ_H_ 12.57 (1H, s, OH-2′), 7.07 (2H, d, *J* = 8.3 Hz, H-2,H-6), 6.55 (d, *J* = 8.3 Hz, H-3,H-5), 5.95 (2H, s, H-3′, H-5′), 3.74 (3H, s, 4′-OCH_3_), 3.25 (2H, t, *J* = 7.8 Hz, H-8), 2.76 (2H, t, *J* = 7.8 Hz, H-8). APT ^13^C NMR (CDCl_3_, 150 MHz) δ_C_ 205.5 (C-9), 166.2 (C-4′), 165.3 (C-2′, C-6′), 155.3 (C-4), 142.3 (C-1), 128.8 (C-2, C-6), 117.5 (C-3,C-5), 105.1 (C-1′), 92.5 (C-3′, C-5′), 56.2 (CH_3_O-4′), 46.1 (C-7), 30.2 (C-8). HRESIMS [M + H]^+^ *m*/*z* 289.1061 (calcd. for C_16_H_17_O_5_, 289.1076).

(−)-Methyllinderatin (**3**). Colourless oil. [α]_D_^20^ = −39.4 (CHCl_3,_ *c* = 0.01). ^1^H NMR (CDCl_3_, 600 MHz) δ_H_ 13.72 (1H, s, OH-2′), 7.27 (1H, d, *J* = 7.6 Hz, H-4), 7.05–7.13 (4H, m, H-2, H-3, H-5, H-6), 6.08 (1H, s, H-5′), 5.45 (1H, s, H-2″), 3.85 (1H, br d, *J* = 10.1 Hz, H-3″), 3.77 (3H, s, CH_3_O-4′), 3.36 (2H, t, *J* = 7.5 Hz, H-7), 3.02 (2H, t, *J* = 7.5 Hz, H-8), 2.16-2.08 (3H, m, H-6″, H-8″), 1.76 (3H, s, H-7″), 1.94 (1H, m, H_α_-5″), 1.80 (1H, m, H-4″), 1.66 (1H, m, H_β_-5″), 0.85 (3H, s, H-9″), 0.79 (3H, s, H-10″). APT ^13^C NMR (CDCl_3_, 150 MHz) δ_C_ 205.5 (C-9), 165.1 (C-2′), 161.7 (C-4′), 159.7 (C6′), 142.1 (C-1), 134.9 (C-1″), 129.0 (C-3, C-5), 128.9 (C-2, C-6), 126.3 (C-4), 126.1 (C-2″), 107.7 (C-3′), 105.1 (C-1′), 92.1 (C-5′), 56.1 (CH_3_O-4′), 46.2 (C-7), 42.9 (C-4″), 35.2 (C-3″), 31.1 (C-6″), 30.9 (C-8), 29.3 (C-8″), 23.6 (C-7″), 23.3 (C-5″), 21.7 (C-10″), 17.8 (C-9″). HRESIMS [M + H]^+^ *m*/*z* 409.2388 (calcd. for C_26_H_33_O_4_, 409.2379).

## 4. Conclusions

Among test botanicals (*n* = 44), four *Piper*-derived extracts showed the most potent inhibitory activity on mycelial growth of *F. oxysporum*, whereas conidia were more susceptible to *P. elongatum* and *T. diversifolia* extracts, having moderate activity. This fact indicated a differential effect of extract components on each fungal form. Extract from leaves of *P. aduncum* exhibited the highest activity on mycelial growth, possibly targeting the cell wall or cell membrane of the fungus. The indirect detection of plausible bioactives through untargeted metabolic profiling, integrating the chemical and antifungal activity against *F. oxysporum* datasets, led to the recognition of three antifungal compounds piperaduncin C (**1**), asebogenin (**2**) and (−)-methyllinderatin (**3**) as the most active compounds. This finding was adequately validated after the isolation of these metabolites and evaluation of the pure isolates against *F. oxysporum* through both assays. This is the first report for these dihydrochalcone-type metabolites against *F. oxysporum.* Therefore, the usefulness of this indirect approach based on HPLC profiles for the identification of antifungals from botanical extracts was confirmed, which extends a rationale for less-time consuming antifungal recognition and lead finding from nature. Additionally, our findings constitute the basis for the development of antifungal agents based on active dihydrochalcone-containing extracts and individual compounds and, consequently, for outlining their potential as promising biorationals to protect plants against this phytopathogen.

## Figures and Tables

**Table 1:** Mycelial growth inhibition (MGI) of test botanical extracts.

#	Plant	BF ^a^	PP ^b^	C ^c^	MGI ^d^ (%)	#	Plant	BF ^a^	PP ^b^	C ^c^	MGI ^d^ (%)
1	*Piper aduncum*	Pi	L	*Pa*L	95.26 ± 0.15 ^A^	24	*Ulex europeus*	F	L	*Ue*L	50.76 ± 0.73 ^P^
2	*Piper elongatum*	Pi	L	*Pe*L	91.31 ± 0.13 ^B^	25	*Bursera simaruba*	Bu	L	*Bs*L	49.74 ± 0.11 ^Q^
3	*Piper elongatum*	Pi	I	*Pe*I	81.26 ± 0.21 ^C^	26	*Croton fragrans*	E	L	*Cf*L	48.43 ± 0.09 ^R^
4	*Piper elongatum*	Pi	S	*Pe*S	76.21 ± 0.11 ^D^	27	*Croton hibiscifolius*	E	L	*Ch*L	47.77 ± 0.23 ^RS^
5	*Anadenanthera peregrina*	F	L	*Ap*L	70.08 ± 0.06 ^E^	28	*Bursera simaruba*	Bu	B	*Bs*B	47.63 ± 0.22 ^S^
6	*Nectandra longifolia*	L	L	*Nl*L	68.80 ± 0.07 ^F^	29	*Galipea longiflora*	Ru	L	*Gl*L	46.91 ± 0.30 ^T^
7	*Raphanus raphanistrum*	Br	L	*Rr*L	64.67 ± 0.13 ^G^	30	*Ficus obtusifolia*	Mo	L	*Fo*L	42.86 ± 0.13 ^U^
8	*Jacaranda obtusifolia*	Bi	L	*Jo*L	62.71 ± 0.34 ^H^	31	*Croton hibiscifolius*	E	S	*Ch*S	41.96 ± 0.43 ^V^
9	*Nectandra acutifolia*	L	L	*Nac*L	62.66 ± 0.15 ^H^	32	*Pyrostegia venusta*	Bi	S	*Pv*S	41.03 ± 0.23 ^W^
10	*Protium heptaphyllum*	Bu	L	*Ph*L	60.66 ± 0.47 ^I^	33	*Baccharis macrantha*	A	L	*Bm*L	40.67 ± 0.13 ^W^
11	*Mimosa pudica*	F	A	*Mp*A	58.48 ± 0.23 ^J^	34	*Phyllanthus salviifolius*	Ph	S	*Ps*S	39.03 ± 0.05 ^X^
12	*Genista monspessulana*	F	F	*Gm*F	56.61 ± 0.18 ^K^	35	*Virola peruviana*	My	L	*Vp*L	38.73 ± 0.09 ^X^
13	*Mimosa nigra*	F	A + F	*Mn*AF	55.69 ± 0.02 ^L^	36	*Trattinnickia rhoifolia*	Bu	L	*Tr*L	36.86 ± 0.18 ^Y^
14	*Anadenanthera peregrina*	F	B	*Ap*B	54.00 ± 0.07 ^M^	37	*Mimosa pudica*	F	L	*Mp*F	27.01 ± 0.07 ^Z^
15	*Bowdichia virgilioides*	F	L	*Bv*L	52.35 ± 0.17 ^N^	38	*Cedrela odorata*	Ml	L	*Cod*L	24.02 ± 0.03 ^AA^
16	*Copaifera officinalis*	F	B	*Co*B	52.25 ± 0.15 ^N^	39	*Senna viarum*	F	L	*Sv*L	22.04 ± 0.15 ^AB^
17	*Hymenaea courbaril*	F	L	*Hc*L	52.21 ± 0.19 ^N^	40	*Tithonia diversifolia*	A	F	*Td*F	19.43 ± 0.31 ^AC^
18	*Croton colombianus*	E	L	*Cc*L	51.96 ± 0.23 ^N^	41	*Virola elongata*	My	L	*Ve*L	16.29 ± 0.04 ^AC^
19	*Mimosa colombiana*	F	A	*Mc*A	51.74 ± 0.16 ^N^	42	*Hymenaea courbaril*	F	S	*Hc*S	14.28 ± 0.18 ^AE^
20	*Baccharis macrantha*	A	F	*Bm*F	51.71 ± 0.11 ^NO^	43	*Cedrela odorata*	Ml	B	*Cod*B	14.23 ± 0.09 ^AE^
21	*Miconia resima*	Mt	L	*Mr*L	51.68 ± 0.11 ^NO^	44	*Tithonia diversifolia*	A	S	*Td*S	12.93 ± 0.18 ^AF^
22	*Nectandra amazonum*	L	L	*Nam*L	51.64 ± 0.05 ^NO^	-	*Dithane*	-	-		99.1 ± 0.06
23	*Cotoneaster pannosus*	Ro	Fr	*Cp*Fr	51.02 ± 0.08 ^OP^	-	*Prochloraz*	-	-		94.4 ± 0.36

^a^ BF = Botanical Family: Pi = Piperaceae; F = Fabaceae; L = Lauraceae; Br = Brassicaceae; Bi = Bignonaceae; Bu = Burseraceae; E = Euphorbiaceae; A = Asteraceae; Mt = Melastomataceae; Ro = Rosaceae; Ru = Rutaceae; Mo = Moraceae; Ph = Phyllanthaceae; My = Myristicaceae; Ml = Meliaceae; ^b^ PP = Plant Part used to prepare the extract: L = Leaves; R = Roots; S = Stems; I = Inflorescences; A = Aerial part; F = Flowers; B = Bark; Fr = Fruits; ^c^ = Code assigned to each extract; ^d^ MGI = Mycelial growth inhibition expressed in percentage. The bioassay involved a final concentration of 0.2% (*w*/*v*) of each extract; Values presented as mean ± standard deviation (*n* = 3). Different superscript uppercase letters indicate significant differences according to the post hoc Tukey test (*p* < 0.05).

**Table 2:** Conidial susceptibility of active extracts.

Plant	Plant Part ^a^	Code ^b^	CGI ^c^ (%)
*Piper elongatum*	L	*Pe*L	83.52 ± 0.78 ^A^
*Tithonia diversifolia*	S	*Td*S	73.95 ± 0.18 ^B^
*Croton hibiscifolius*	S	*Ch*S	47.60 ± 0.11 ^C^
*Piper elongatum*	S	*Pe*S	24.01 ± 0.15 ^D^
*Piper aduncum*	L	*Pa*L	21.13 ± 0.02 ^E^
*Tithonia diversifolia*	F	*Td*F	15.36 ± 0.06 ^F^
*Dithane*	-	-	97.36 ± 0.02
*Prochloraz*	-	-	53.62 ± 0.26

^a^ Plant Part used to prepare the extract: L = Leaves; S = Stems; F = Flowers; ^b^ C = Code assigned to each extract; ^c^ CGI = Conidial germination inhibition expressed in percentage. The bioassay involved a final concentration of 0.1% (*w*/*v*) of each extract. Values expressed as mean ± standard deviation (*n* = 4). Different superscript uppercase letters indicate significant differences according to the Tukey test (*p* < 0.05).

**Table 3:** Antifungal activity of isolated compounds from *Piper* species.

	Piperaduncin C (1)	Asebogenin (2)	(−)-Methyllinderatin (3)
MGI IC_50_ (µM) ^a^	38.2 (31.5–44.8)	25.6 (23.5–29.3)	689.2 (623.8–744.9)
CGI IC_50_ (µM) ^b^	>1000	>1000	22.3 (17.8–29.7)

^a^ Half maximal inhibitory concentration (IC_50_ in μM) for the inhibition of *F. oxysporum* mycelial growth. ^b^ Half maximal inhibitory concentration (IC_50_ in μM) for the inhibition of *F. oxysporum* conidial germination. Data are expressed as best-fit value (95% confidence interval) (*n* = 4).

## Data Availability

The data that support the findings of this study are available from the corresponding author upon request.
